# Oxygen delivery systems for adults in Sub-Saharan Africa: A scoping review

**DOI:** 10.7189/jogh.11.04018

**Published:** 2021-05-08

**Authors:** Neelima Navuluri, Maria L Srour, Peter S Kussin, David M Murdoch, Neil R MacIntyre, Loretta G Que, Nathan M Thielman, Eric D McCollum

**Affiliations:** 1 Division of Pulmonary, Allergy, and Critical Care, Department of Medicine, Duke University School of Medicine, Durham, North Carolina, USA; 2 Duke Global Health Institute, Duke University, Durham, North Carolina, USA; 3 Division of Pulmonary, Allergy, and Critical Care, Department of Medicine, Indiana University School of Medicine, Indianapolis, Indiana, USA; 4 Division of Infectious Diseases, Department of Medicine, Duke University School of Medicine, Durham, North Carolina, USA; 5 Global Program in Respiratory Sciences, Eudowood Division of Pediatric Respiratory Sciences, Department of Pediatrics, Johns Hopkins School of Medicine, Baltimore, Maryland, USA

## Abstract

**Background:**

Respiratory diseases are the leading cause of death and disability worldwide. Oxygen is an essential medicine used to treat hypoxemia from respiratory diseases. However, the availability and utilization of oxygen delivery systems for adults in sub-Saharan Africa is not well-described. We aim to identify and describe existing data around oxygen availability and provision for adults in sub-Saharan Africa, determine knowledge or research gaps, and make recommendations for future research and capacity building.

**Methods:**

We systematically searched four databases for articles on April 22, 2020, for variations of keywords related to oxygen with a focus on countries in sub-Saharan Africa. Inclusion criteria were studies that included adults and addressed hypoxemia assessment or outcome, oxygen delivery mechanisms, oxygen availability, oxygen provision infrastructure, and oxygen therapy and outcomes.

**Results:**

35 studies representing 22 countries met inclusion criteria. Availability of oxygen delivery systems ranged from 42%-94% between facilities, with wide variability in the consistency of availability. There was also wide reported prevalence of hypoxemia, with most studies focusing on specific populations. In facilities where oxygen is available, health care workers are ill-equipped to identify adult patients with hypoxemia, provide oxygen to those who need it, and titrate or discontinue oxygen appropriately. Oxygen concentrators were shown to be the most cost-effective delivery system in areas where power is readily available.

**Conclusions:**

There is a substantial need for building capacity for oxygen delivery throughout sub-Saharan Africa. Addressing this critical issue will require innovation and a multi-faceted approach of developing infrastructure, better equipping facilities, and health care worker training.

Respiratory diseases are the leading cause of death and disability worldwide. Oxygen is an essential, life-saving medical therapy that has been used to treat respiratory disease since the late 1800s [1,2]. It is used to treat both acute and chronic conditions which result in hypoxemia, which is an abnormally low concentration of oxygen in the blood. For adult patients with chronic hypoxemia from primary lung disease or heart failure, long term oxygen therapy is a well-established cornerstone of management, improving survival and quality of life [3,4].

Oxygen has been listed as one of the World Health Organization’s (WHO) Essential Medicines since the first online edition in 2002, but only for anesthesia. Hypoxemia was added as an indication only recently in 2017 [5,6]. The simplest way to identify patients who are hypoxemic is by measuring oxygen saturation of the blood with a pulse oximeter, which uses infrared light refraction to non-invasively measure the percentage of oxygen in red blood cells. Supplemental oxygen can be provided via cylinders (gas or liquid), oxygen concentrators, or larger oxygen plants, each of which have unique advantages and disadvantages depending on environment and resource infrastructure. WHO guidelines on the clinical use of oxygen in children and technical specifications for oxygen equipment are available [7,8]. These highlight the need for pulse oximetry, appropriate clinical evaluation and management of underlying etiology, and basic administration standards. They notably do not specify guidelines for adults.

Despite widespread recognition of the importance of oxygen therapy for treatment of hypoxemia, its use and implementation remain inadequate in much of the world. Specifically, there is limited data about hypoxemia recognition and oxygen provision across sub-Saharan Africa (SSA), with most existing data focusing on acute oxygen needs among children and neonates. In order to design interventions and implementation efforts, a better understanding of the existing ecosystem is required, especially as it relates to adult populations.

In this scoping review, we aimed to identify and describe existing data around oxygen availability and provision for adults in SSA in order to determine areas of research or knowledge gaps on this topic and make recommendations for future research and capacity building. We hypothesized that the literature on adults would be limited as compared to pediatric populations, that what did exist would highlight the lack of availability, usage, and utilization of oxygen delivery systems for adults, and would identify key barriers of oxygen usage and opportunities for future research and capacity building.

## METHODS

We used the Arksey and O’Malley methodological framework, along with Levac’s recommendations for each stage of the framework, to perform a scoping review of what is known about oxygen delivery systems in SSA [9,10]. A review framework was prepared to develop the overall study protocol including identifying the research question, searching for relevant studies, selecting studies, charting the data, and collating, summarizing, and reporting the results.

### Identifying the research question

The central research question of this scoping review is: what is known in existing literature about oxygen provision to adult patients with hypoxemia in SSA, what knowledge and research gaps exist, and what recommendations for future research and capacity building can be made?

### Search strategy and selection criteria

We performed a systematic search of online databases (PubMed, EMBASE, African Index Medicus, and Web of Science) on April 22, 2020, with the assistance of a medical librarian. All articles from 1970-2020 were included. The review was conducted applying the search words “Africa,” “oxygen inhalation therapy,” “oxygen/supply and distribution,” “oxygen/therapeutic use,” and “oxygen” with “domiciliary,” “home,” “therapy,” “concentrator,” “tank,” “cylinder,” “delivery,” “distribution,” or “supply,” as the Medical Subject Heading (MeSH) headings (**Table 1**).We included any study which included adults and addressed any of the following areas: hypoxemia assessment or outcome, oxygen delivery mechanisms, oxygen availability, oxygen provision infrastructure, and oxygen therapy and outcomes. Basic science studies, case reports, studies focusing only on neonates, children, or fetal outcomes among pregnant women, hyperbaric oxygen therapy, mechanical ventilation or oxygen in the setting of anesthesia, studies based wholly outside of SSA, and those for which no full article could be found were excluded. One investigator reviewed the titles and abstract of the studies for inclusion and an additional investigator assisted in full-text review for final inclusion. These two investigators also charted, collated, and summarized the data.

**Table 1:** Search terms

Set	Terms
#1	“Africa”[Mesh]. OR Africa[all fields]. OR Algeria[all fields]. OR Angola[all fields]. OR Benin[all fields]. OR Botswana[all fields]. OR “Burkina Faso”[all fields]. OR Burundi[all fields]. OR “Cabo Verde”[all fields]. OR “Cape Verde”[all fields]. OR “Central African”[all fields]. OR Chad[all fields]. OR Comoros[all fields]. OR Congo[all fields]. OR “Cote d Ivoire”[all fields]. OR “Cote dIvoire”[all fields]. OR Congo[all fields]. OR Djibouti[all fields]. OR Egypt[all fields]. OR ” Guinea”[all fields]. OR Eritrea[all fields]. OR Ethiopia[all fields]. OR Gabon[all fields]. OR Gambia[all fields]. OR Ghana[all fields]. OR Guinea[all fields]. OR Kenya[all fields]. OR Lesotho[all fields]. OR Liberia[all fields]. OR Libya[all fields]. OR Libyan[all fields]. OR Madagascar[all fields]. OR Malawi[all fields]. OR Mali[all fields]. OR Mauritania[all fields]. OR Mayotte[all fields]. OR Morocco[all fields]. OR Mozambique[all fields]. OR Namibia[all fields]. OR Niger[all fields]. OR Nigeria[all fields]. OR Rwanda[all fields]. OR Sahel[all fields]. OR “Sao Tome and Principe”[all fields]. OR Senegal[all fields]. OR “Sierra Leone”[all fields]. OR Somalia[all fields]. OR “South Africa”[all fields]. OR “South Sudan”[all fields]. OR Sudan[all fields]. OR Swaziland[all fields]. OR Tanzania[all fields]. OR Togo[all fields]. OR Tunisia[all fields]. OR Uganda[all fields]. OR Sahara[all fields]. OR Zambia[all fields]. OR Zimbabwe[all fields].
#2	“Oxygen Inhalation Therapy”[Mesh]. OR “Oxygen/supply and distribution”[Mesh:NoExp]. OR “Oxygen/therapeutic use”[Mesh:NoExp]. OR (oxygen[tiab]. AND (domiciliary[tiab]. OR home[tiab]. OR therapy[tiab]. OR delivery[tiab]. OR distribution[tiab]. OR supply[tiab]. OR concentrator*[tiab]. OR cylinder*[tiab]. OR tank*[tiab].))
#3	#1 AND #2
#4	(randomized controlled trial[pt]. OR controlled clinical trial[pt]. OR randomized[tiab]. OR randomised[tiab]. OR randomization[tiab]. OR randomisation[tiab]. OR placebo[tiab]. OR randomly[tiab]. OR trial[tiab]. OR study[tiab]. OR groups[tiab]. OR Clinical trial[pt]. OR “clinical trial”[tiab]. OR “clinical trials”[tiab]. OR “evaluation studies”[Publication Type]. OR “evaluation studies as topic”[MeSH Terms]. OR “evaluation study”[tiab]. OR evaluation studies[tiab]. OR “intervention study”[tiab]. OR “intervention studies”[tiab]. OR “case-control studies”[MeSH Terms]. OR “case-control”[tiab]. OR “cohort studies”[MeSH Terms]. OR cohort[tiab]. OR “longitudinal studies”[MeSH Terms]. OR “longitudinal”[tiab]. OR longitudinally[tiab]. OR “prospective”[tiab]. OR prospectively[tiab]. OR “retrospective studies”[MeSH Terms]. OR “retrospective”[tiab]. OR observational[tiab]. OR “follow up”[tiab]. OR “comparative study”[Publication Type]. OR “comparative study”[tiab]. OR systematic[subset]. OR “meta-analysis”[Publication Type]. OR “meta-analysis as topic”[MeSH Terms]. OR “meta-analysis”[tiab]. OR “meta-analyses”[tiab].) NOT (Editorial[ptyp]. OR Letter[ptyp]. OR Case Reports[ptyp]. OR Comment[ptyp].) NOT (animals[mh]. NOT humans[mh].)
#5	#4 AND #3

### Charting the data, summarizing and reporting

We used a data collection form adapted from the Cochrane Effective Practice and Organization of Care group [11]. For each included study, we extracted data on study design, country or region of study, sample size, oxygen delivery device and patient interface, power source, population setting, facility, measured outcomes, and interventions where available.

The data were recorded in a series of tables, enabling repeated exploring of themes. After an iterative process of data extraction, recording, and thematic review, we aggregated studies into major themes and then collated the data to help describe the existing knowledge around oxygen availability and provision and analyze for key knowledge and research gaps.

### Patient and public involvement

No patients nor members of the public were involved in the review.

## RESULTS

### Description of studies

**Figure 1:**
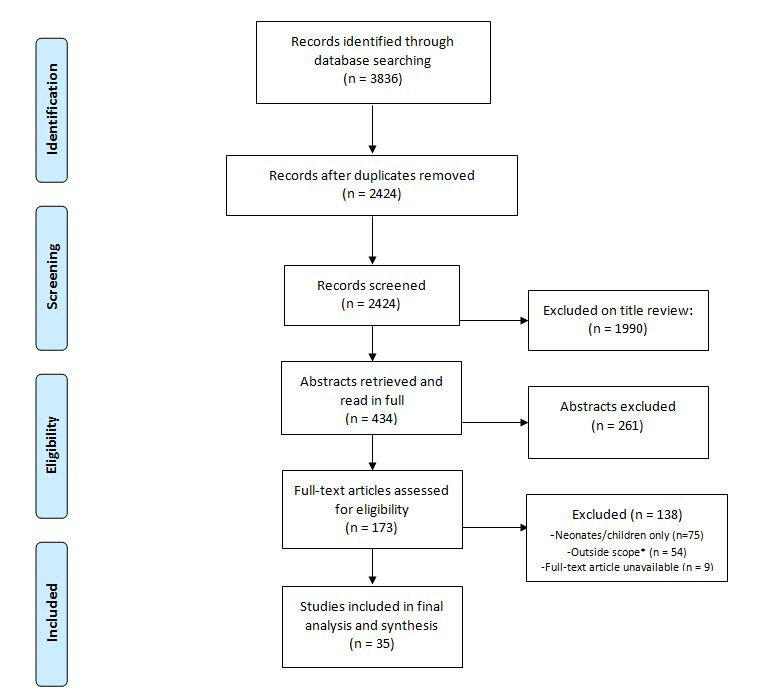
PRISMA flowchart detailing study selection. *Did not describe current state of oxygen availability, provision and/or oxygen delivery mechanisms or were studies based entirely outside of SSA.

Of 2424 papers screened, 173 full-text articles were assessed for eligibility (**Figure 1**). We ultimately included 35 articles describing 34 different studies.

**Figure 2:**
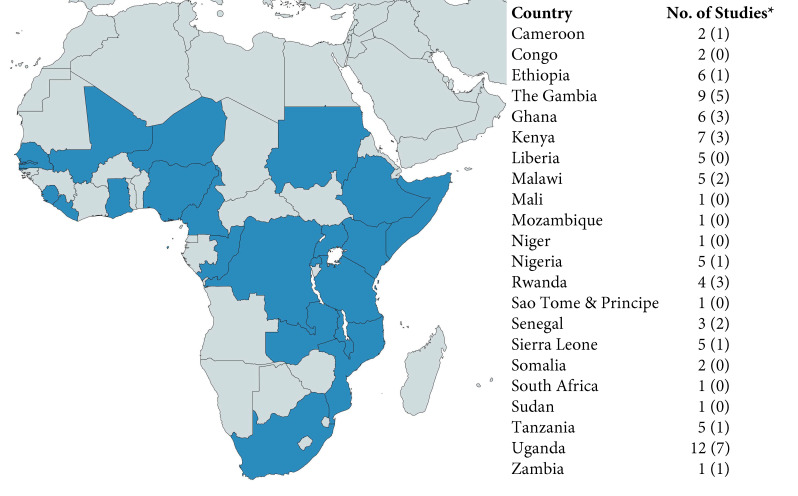
Countries represented in available studies. *First number indicates number studies in total, including studies which included multiple sites. Number in parenthesis indicates number of single-site studies.

A total of 22 SSA countries were represented in the studies (**Figure 2**), with 5 studies including data from multiple countries. Studies were published between 1995 and 2020 with data collection ranging from 1995 to 2017.

We identified three key themes among these articles, each with multiple sub-themes about the use of oxygen therapy in SSA (**Table 2** and **Table 3**). These themes are oxygen availability, infrastructure, and usage (n = 26), hypoxia assessment and clinical understanding and management (n = 24), and cost and cost-effectiveness (n = 4), and each are elaborated upon below. Eight articles contained two themes.

**Table 2:** Oxygen availability, infrastructure, and usage and cost and cost-effectiveness

Author, year	Country	Facility type and setting	Data source	Key findings
**Adipa, 2015 [**12**]**	Ghana	Korle Bu Teaching Hospital – Emergency Department and ICU	Face to face interviews with health care workers (HCWs)	Oxygen was readily available via cylinders, but cumbersome tanks often had to be moved across wards for patient access.
**Albutt, 2018 [**13**]**	Uganda	17 public hospitals	Surgical Assessment Tool (SAT) developed by the Program in Global Surgery and Social Change and the WHO	Oxygen was available more than half the time at 68.8% of facilities while continuous pulse oximetry was always available in the operating room in only 62.5% of hospitals.
**Albutt, 2019 [**14**]**	Uganda	16 private and private not-for-profit hospitals	WHO Tool for Situational Analysis to Assess Emergency and Essential Surgical Care (TSAAEESC)	93.8% of hospitals had oxygen but only 37.5% of had it for more than 25% of the time.
**Belle, 2010 [**15**]**	Ethiopia, The Gambia, Ghana, Kenya, Liberia, Malawi, Mali, Nigeria, Sierra Leone, Sao Tome & Principe, Tanzania, Uganda	231 health centers and hospitals; 23% private, 38% primary care, 31% district or regional and 8% general hospitals	WHO TSAAEESC	43.8% of hospitals had a consistently available oxygen source; 31.4% had intermittent availability. Oxygen cylinders, concentrators and face masks and tubing were readily available in less than 35% of hospitals. Electricity and generator supply were similarly sporadic.
Bradley, 2015* [16**]**	The Gambia	Biomedical Engineering Department at the Medical Research Council Unit	Retrospective analysis of electronic maintenance records for 27 oxygen concentrators	The majority of concentrator faults were repairable for less than US$10, with the average costs of the most common repairs - filter and check valve replacements - US$4.53 and US$6.80 respectively. Median cost of repairs was US$9.44 with a maximum of US$573. The authors predict a seven year lifespan for concentrators in low resource settings with a US$15 per machine-year of service repair cost.
Bradley, 2016* [17**]**	The Gambia	42 bed hospital in the Medical Research Council Unit	Assessment of oxygen concentrator function and user feedback	The hospital system saved 51% of oxygen costs by using concentrators which amounted to US$45 000 over the course of 8 y.
**Burssa, 2017 [**18**]**	Ethiopia	29 facilities throughout Ethiopia	Ministry of Health Assessment	Continuous oxygen supply was not available in 33% of facilities and 59% had interrupted electricity. As part of their Safe Surgery initiative, 2 oxygen plants were built at referral hospitals.
**Calderon, 2019 [**19**]**	Uganda	Jinja Regional Referral Hospital	Prototype Assessment	A low-pressure reservoir storage system was able to deliver oxygen in 56% of power outage minutes and cover over 99% of power outage events.
**Desalu, 2011 [**20**]**	Nigeria	68 tertiary care hospitals	Cross-sectional survey	In the studied tertiary care centers, 52.9% reported having a standard oxygen delivery system. Under 40% had pulse oximeters.
**Evans, 2012 [**21**]**	Malawi	Queen Elizabeth Central Hospital (QECH) – 1200 bed public, teaching hospital in Southern Malawi	Cross-sectional study of adult medical inpatients and oxygen provision over 24 h	8concentrators were present, but only 4 functioned appropriately. Three had oxygen flow rates <60% of indicated; 1 did not function at all.
**Hill, 2009 [**22**]**	The Gambia	12 health facilities – 5 government referral hospitals, 7 health centers	Standardized WHO questionnaire	6 of the 12 facilities surveyed had available oxygen supplies (cylinders and concentrators). The government central referral hospitals generally had good reliability of supply whereas large health centers did not.
**Howie, 2008 [**23**]**	The Gambia	Royal Victoria Teaching Hospital (tertiary referral center)	Interviews with HCWs	Oxygen concentrators were donated from North America but required a different electrical frequency so were difficult to use. Very little training was given to providers and eventually the supply of concentrators went unused.
Howie, 2009* [24**]**	The Gambia	11 public hospitals	Health needs assessment framework	Concentrators have significant advantage compared to cylinders where power is reliable. In other settings cylinders preferred if transport is feasible. Cylinder costs are influenced by leakage whereas concentrator costs are affected by cost of power. Only 2 of 12 facilities in Gambia were suitable for concentrators over cylinders.
**Kouo-Ngamby, 2015 [**25**]**	Cameroon	12 public hospitals	WHO TSAAEESC	8 of the 12 hospitals had oxygen cylinder supply, but only 4 of the 12 had reliable oxygen concentrators. The greatest equipment needs were demonstrated in facilities providing lower tiers of care.
**Kushner, 2010 [**26**]**	Tanzania, Sierra Leone, Liberia, The Gambia	132 district hospitals in 30 LMICs	WHO TSAAEESC	No country reported 100% of facilities having continuous water, electricity and oxygen supplies. Oxygen was never available in 46% of facilities.
**LeBrun, 2014 [**27**]**	Ethiopia, Liberia, Rwanda, Uganda	78 district hospitals	WHO TSAAEESC	Approximately 80% of hospitals had a reliable oxygen source in the operating theater (OT) while 59% had pulse oximeters in each OT and 33% had them in surgical recovery rooms. Many of the hospitals surveyed reported power outages or interruptions.
**Nyarko, 2016 [**28**]**	Ghana	23 health facilities of various care levels; all received some if not all funding from the Ghanaian government	Facility-based survey to assess WHO- Package of Essential Noncommunicable Disease Interventions	None of the community-based health care facilities had functional oxygen cylinders or pulse oximeters while district and regional hospitals all did. Access to medications and medical equipment improved with increased levels of care.
**Ologunde, 2014 [**29**]**	Congo, Ethiopia, The Gambia, Ghana, Kenya, Liberia, Malawi, Niger, Nigeria, Sierra Leone, Somalia, Uganda, Tanzania	719 health facilities	WHO TSAAEESC	Oxygen was available in 73.3% of the facilities surveyed. At facilities that offer cesarean delivery, 78% had access to oxygen whereas only 21% of facilities that did not offer the procedure had available oxygen. The survey did not distinguish between cylinders and concentrators.
**Otiangala, 2020 [**30**]**	Kenya	11 rural health facilities	Key informant interviews	Of 11 facilities surveyed, 82% had at least one cylinder and at least one concentrator. A back-up generator was available at 64% of facilities. The study also found a high case fatality rate in hypoxemic patients with an upward trend in mortality in those who experienced interruptions in therapeutic oxygen supply.
**Opio, 2014 [**31**]**	Uganda	Kitovu Hospital – 220 bed private not-for-profit regional hospital	Prospective data	This study compared prospective data from Uganda to retrospective data from Canada to compare in-hospital mortality in patients with similar admission characteristics. It noted that there was only 1 oxygen concentrator at the hospital in Uganda.
**Ouedraogo, 2018 [**32**]**	Senegal	All formal sector health facilities in the country	Senegal Service Provision Assessments	52% of health care facilities do not have access to regular uninterrupted electricity. Most of these facilities are connected to the central grid with only 18% of facilities using a generator or solar supply. 11% of facilities with oxygen concentrators do not have the electricity to power them.
**Penoyar, 2012 [**33**]**	Tanzania	48 public hospitals	WHO TSAAEESC	Of the facilities surveyed, which together serve 46% of the Tanzanian population, 42% had consistent access to oxygen delivery, most of which used concentrators. Only 37.5% had reliable running water and electricity. A total of 6 functional pulse oximeters were located across all 48 facilities.
Rassool, 2017* [34**]**	Uganda	Mbarara Regional Hospital (referral hospital)	Field testing of a low-pressure oxygen storage system	A low-pressure oxygen storage system designed by a team in Australia was able to provide continuous oxygen supply to a simulated patient without interruption for 30 d. The estimated cost of the system was US$460. The main drawback noted was the large amount of space the system occupies.
**Rudd, 2017 [**35**]**	Uganda	Bwindi Community Hospital – a private, 112-bed, rural hospital	Prospective observational single cohort study	Of 199 patients admitted to the hospital during the study period, 62 met SIRS criteria and were enrolled in the study. In the adult population, 44% of patients hypoxic to <94% O_2_ were treated with oxygen therapy; 100% of those hypoxic to <90% were. Only 1 of the patients studied died in the hospital while 92% of patients went home in improved condition.

*Article also addresses cost and/or cost-effectiveness.

**Table 3:** Hypoxemia assessment and clinical understanding and management articles

Author, year	Country	Facility type and setting	Data source	Key findings
**Adipa, 2015 [**12**]**	Ghana	Surgical Medical Emergency and Cardiothoracic Intensive Care Unit at Korle Bu Teaching Hospital	Face to face interviews with health care workers (HCWs)	Nursing education on oxygen therapy was inadequate and a knowledge gap exists. Nurses require more training in assessing oxygen needs and administering therapy appropriately.
**Aston, 2019 [**36**]**	Malawi	Queen Elizabeth Central Hospital (QECH) - 1200 bed public, teaching hospital in Southern Malawi	Prospective observational study	Hypoxemia (SpO_2_<90%) was strongly associated with 30-d mortality among 459 patients hospitalized with community-acquired pneumonia (CAP). Authors comment that oxygen provision is an obvious strategy to improve CAP outcomes.
**Dickson, 2018 [**37**]**	Sierra Leone	UK military Ebola treatment center	Retrospective analysis of clinical charts	The lowest oxygen saturations recorded in fatal cases were lower than cases that survived. Oxygen was administered to 18/41 Ebola patients via concentrators and 16 of those 18 patients died.
**Evans, 2012 [**21**]**	Malawi	QECH	Cross-sectional study of adult medical inpatients and oxygen provision over 24 h	14 of 144 (9%) patients studied had SpO_2_<90% and met criteria for oxygen supplementation, but only 4 (29%) received O_2_ therapy.
**Foran, 2010 [**38**]**	Zambia	Kapiri District Hospital - 60 bed government run district hospital	Cross-sectional study of inpatients	109 adults were assessed, of whom 9% were hypoxemic (SpO_2_<90%)
**Hesse, 1995 [**39**]**	Ghana	Korle-Bu Teaching Hospital	Cross-sectional survey of 72 first-year doctors	25% of doctors surveyed were demonstrated to manage asthma appropriately. 28% of doctors used oxygen for mild to moderate asthma, and 74% used it for severe asthma. Some doctors were not aware there was some degree of hypoxemia even with mild asthma
**Kabeza, 2012 [**40**]**	Rwanda	Kigali University Teaching Hospital	Prospective observational study of 125 patients undergoing abdominal surgery	24% of patients transferred to PACU were hypoxemic (SpO_2_<90%) and of those, 27% were transferred without supplemental O_2_. 50% of patients were hypoxemic at least once during the study period.
**Mwita, 2016 [**41**]**	Kenya	Thika Level 5 hospital in Kiambu County in Central Kenya	Retrospective clinical audit	This study assessed the compliance with traumatic brain injury (TBI) management criteria, one of which is oxygen therapy. Only 21% of patients 13yrs or older (mean age 29.8) with TBI were administered oxygen as indicated. The authors speculate that lack of availability is a significant factor.
**Opio, 2014 [**31**]**	Uganda	Kitovu Hospital – 220 bed private not-for-profit regional hospital	Prospective data	This study compared prospective data from Uganda to retrospective data from Canada to compare in-hospital mortality in patients with similar admission characteristics. It noted 3.9% of patients were on supplemental oxygen vs 31.2% of patients in Canada.
**Rudd, 2017 [**35**]**	Uganda	Bwindi Community Hospital – a private, 112-bed, rural hospital	Prospective observational single cohort study	Of 199 patients admitted to the hospital during the study period, 62 met SIRS criteria and were enrolled in the study. In the adult population, 44% of patients hypoxic to <94% O_2_ were treated with oxygen therapy; 100% of those hypoxic to <90% were. Only one of the patients studied died in the hospital while 92% of patients went home in improved condition.
**Sani, 2017 [**42**]**	Senegal, Nigeria, Cameroon, Ethiopia, Kenya, South Africa, Mozambique, Uganda, Sudan	12 cardiology centers	Prospective multicenter observational survey	Assessed changes in signs and symptoms of heart failure over the course of hospitalization. Oxygen saturation was predictive of death or heart failure exacerbation through day 60.
**Scott, 2017 [**43**]**	Rwanda	National ambulance system – Service d’Aide Médicale Urgente (SAMU)	Prospective Quality Improvement (QI) Study	Implemented monthly presentations to the ambulance service and assessed quality metrics including supplementary oxygen for hypoxia. Use of supplemental oxygen improved from 75% pre-intervention to 92% post-intervention.
**Sutherland, 2019 [**44**]**	Rwanda	University Teaching Hospital of Kigali Emergency Department	QI Study	12.1% of 1765 patients screened were hypoxemic. 81.3% of patients were either under- or over-treated with oxygen. An intervention that provided didactics and pulse oximetry to providers helped improve clinician's knowledge scores, oxygen provision and titration, and decreased tank use per day. Results persistent at 4 weeks and 12 weeks.
**Toure, 2000 [**45**]**	Senegal	Dakar University Hospital Center Cardiology Department	Retrospective Cross Sectional	30 of 34 (88.8%) patients with cor pulmonale were hypoxemic.
**Worodria, 2018 [**46**]**	Uganda	Mulago Hospital	Secondary analysis of established cohort of adult patients hospitalized with lower respiratory tract infection	10.6% of 1887 patients had severe hypoxemia (SpO_2_<90%) on RA; hypoxemia was associated with in-hospital and post-hospital mortality (adjusted OR of 2.75 and 2.09 respectively).

### Oxygen availability, infrastructure, and usage

Several of the reviewed studies used surveys to assess the available equipment in health care settings throughout SSA. These surveys showed a lack of basic equipment such as pulse oximeters and oxygen delivery systems, eg, cylinders or concentrators. Availability of oxygen delivery systems ranged from 42%-94% between facilities, with wide variability in the consistency of availability [13-15,18,20,22,24,25,28,30,33]. For example, the highest reported availability was in a study of private and public facilities in Uganda which showed that 15 of 16 facilities had access to oxygen at least half of the time. However, six of those hospitals lacked access to oxygen more than 25% of the time and one hospital never had access to oxygen [14]. The availability of pulse oximetry was significantly more limited, ranging from 0%-64% of facilities assessed [20,27,33]. This suggests that while facilities may have oxygen available, their ability to accurately identify patients who may need oxygen and titrate the amount delivered appropriately is limited. These limitations are especially true for public facilities and those providing lower tiers of care [13,14,28].

Three studies assessed infrastructure in low- and middle-income countries (LMICs) across the world; data specific to SSA was extracted from these studies and demonstrate similar findings [26,27,29]. These studies were focused on surgery and anesthesia capacity, but included assessments of the facilities at large and thus were included in this review. Kushner et al found 46% of facilities across eight LMICs (four of which were in SSA – Tanzania, Sierra Leone, Liberia, and The Gambia) never had oxygen available, 33% had it sometimes available, and 21% had it always available [26]. Likewise, a cross-sectional survey assessing cesarean-section delivery capacity across 26 LMICs (13 in SSA – Congo, Ethiopia, The Gambia, Ghana, Kenya, Liberia, Malawi, Niger, Nigeria, Sierra Leone, Somalia, Uganda and Tanzania) found that 21% of facilities performing cesareans reported not having a reliable supply of oxygen and that 26% of those referring out did not have any supply [29]. An additional study which compared in-hospital mortality among inpatients between an Ugandan hospital and Canadian hospital noted that there was only one oxygen concentrator available in a large regional hospital in Uganda [31].

Issues in oxygen availability extend beyond the availability of the oxygen delivery systems themselves. Delivering oxygen from a cylinder or concentrator to a patient requires basic equipment such as tubing to connect the system to a patient delivery device such as a face mask or nasal prongs. A notable multi-SSA country assessment demonstrated that not only do less than half of the facilities report access to an oxygen source, but that only 34.3% had at least one face mask and tube set available [15]. Furthermore, for oxygen concentrators to function, electricity must be readily available. This is a major issue for many facilities, with only 35%-68% having electricity fully available [15,27,32]. Backup power generators are often utilized in areas where consistent electricity may be lacking, but many facilities do not have access to functioning generators, relying instead on solar power [15,27,30,32]. Even in places where oxygen concentrators and power are available, many do not function properly or provide the indicated amount of oxygen [21,23].

There are promising strides in systematically determining the best approach to providing oxygen and developing innovative oxygen delivery and storage systems in under-resourced areas. Oxygen concentrators have been demonstrated to be much more cost-effective than cylinders for oxygen delivery without sacrificing medical benefit [24]. Replacing cylinders with oxygen concentrators and addressing the issue of reliable power by installing an uninterrupted power supply has been shown to be easy to maintain and cost-effective [17]. Oxygen storage systems or reservoirs, which store oxygen in low-pressure devices, have been effective in maintaining flow regardless of interruption in power supply for up to 30 days and reducing the number of oxygen outage events [19,34]. While there are limitations to large-scale implementation of these types of reservoirs including clinical trial data, space requirements, and significant need for personnel education, innovations such as these will be imperative in overcoming the “energy poverty” that challenges SSA [32].

There are also issues in the way in which concentrators are provided. In 2000, a group of North American philanthropists donated more than 20 oxygen concentrators to the Royal Victoria Teaching Hospital, The Gambia’s tertiary referral hospital. However, within weeks, none of the concentrators remained in use. A technical and qualitative assessment found that the donation process was flawed, the receiving personnel were not adequately trained in the use or maintenance of the machines, and the electrical frequency of the devices was different than the hospital’s electrical supply and would never have worked, even with a transformer [23].

### Hypoxemia assessment and clinical understanding and management

An understanding of the prevalence of hypoxemia is critical in ensuring that the supply of oxygen in areas where it may be available is truly meeting the demand. Furthermore, medical staff and providers should have a clear understanding of the indications of oxygen, the appropriate route and titration of oxygen therapy, and when to discontinue it. Ten studies addressed the question of prevalence of hypoxemia and demonstrated variability in the assessment of hypoxemia across institutions and countries, as well as the lack of generalized data with most focusing on very specific populations. An additional five provided insight on the understanding of oxygen therapy and management of hypoxemic patients in SSA.

Overall prevalence of hypoxemia, as measured by handheld pulse oximetry among adults in a district hospital in Zambia was 9%, among adult inpatients in Malawi was 9%, and among adults in the emergency department at a teaching hospital in Rwanda was 12.1% [21,38,44]. The study comparing a Ugandan hospital and Canadian hospital assessed oxygen saturation but did not remove oxygen supplementation at the time of measurement, making their values difficult to interpret [31].

Five studies assessed hypoxemia among specific populations, ranging from those with cor pulmonale to sepsis to Ebola Virus Disease. The heterogeneity in patient populations makes it difficult to discern the overall need in various settings, but prevalence ranged from 11%-89% [35,37,40,45,46]. Several of these studies demonstrated high mortality among those with hypoxemia and an additional two studies focused specifically on hypoxemia as a risk factor for mortality [36,42]. For example, among hospitalized patients who presented with lower respiratory tract infection and cough at Mulago Hospital in Uganda, 10.6% had an oxygen saturation of <90%, and hypoxemia was associated with both in-hospital and two-month mortality [46]. Among patients with Ebola Virus Disease in West Africa, 18 of 41 (44%) patients required and received supplemental oxygen via concentrators, and 16 of 18 (89%) died [37]. The highest prevalence was seen among 34 patients with chronic cor pulmonale in Senegal where 88.8% were hypoxemic [45]. Unsurprisingly, oxygen saturation on admission was found to be a predictor of mortality among patients with heart failure across sub-Saharan Africa,[42] adults admitted for community-acquired pneumonia in Malawi, [36] and associated with mortality among hospitalized patients managed for chronic obstructive pulmonary disease (COPD) in Nigeria [47].

In order to ensure understanding and management of hypoxemia, there is a critical need for equipment such as pulse oximetry, more training in identifying and responding to hypoxemia with oxygen, and standardized protocols to guide initiation, titration, and discontinuation of oxygen therapy. Even when hypoxemia was identified, oxygen was not always provided or used appropriately with only 27%-29% of patients with hypoxemia receiving oxygen in two different studies [21,40]. Similarly, compliance with oxygen therapy in traumatic brain injury patients was found to be “dismal” [41]. An assessment of first year doctors in Ghana found that 28% prescribed oxygen for mild to moderate asthma and 74% for severe asthma, with many unaware that there was some degree of hypoxemia even with mild asthma [39]. A qualitative assessment in Ghana found nurses had a lack of knowledge about the appropriate amount of oxygen to provide and when to discontinue oxygen, and they strongly desired standardized protocols [12].

Efforts are being made to improve these metrics as highlighted by a quality improvement project in which daily team meetings and monthly feedback sessions improved supplemental oxygen administration for hypoxia from 75% to 92% among pre-hospital care providers in Rwanda [43].

### Cost and cost-effectiveness

Four articles addressed the cost and cost-effectiveness of various oxygen delivery mechanisms, three of which were based on projects in The Gambia [16,17,24,34]. Oxygen concentrator systems with an uninterruptable power supply could save up to 51% of oxygen supply costs as compared to cylinders, amounting to a total cost of only US$45 000 over the course of eight years [17]. However, the cost-advantage only applies in areas where power is reliable, which as previously described is a major barrier for many facilities [24]. Fortunately, innovations such as the low-pressure oxygen storage system tested in Uganda cost as little as US$460 and generate little extra electricity costs [34]. Concentrators can also be properly maintained in resource-limited settings with most concentrator faults repairable for less than US$10 on average [16].

## DISCUSSION

Our scoping review of available studies reveals that there is a widespread lack of access to and infrastructure for oxygen delivery systems across SSA, and that while this is a relatively understudied area with limited literature, there are several key opportunities to address this issue. It is noted that the majority of studies on oxygen therapy in SSA focus on neonates and children, which is understandable given the high mortality from pneumonia for children under five years. However, respiratory diseases remain the leading cause of death and disability in the world, with diseases such as COPD, asthma, pulmonary hypertension, lung cancer, and tuberculosis killing millions of adults annually. Although efforts are being made to address prevention of these diseases, huge strides are needed to reduce the burden of these diseases and resulting mortality among adults [1]. Availability of and access to oxygen therapy to treat patients with acute or chronic hypoxemia from these diseases is paramount.

Our review of the available studies involving adults demonstrates a dire lack of access to oxygen delivery systems across SSA and that most facilities are ill-equipped to identify adult patients with hypoxemia, provide oxygen to those who need it, and titrate or discontinue oxygen appropriately. Data are limited to mainly surveys, assessments, and observational studies, which cannot be validated, and the simple existence of an oxygen delivery system cannot be directly correlated with patient outcomes. Yet hypoxemia is still associated with significant mortality in many adult populations, and the ability to address it is limited in many care settings. Together, our findings highlight a number of important limitations and opportunities in addressing a critical health issue throughout SSA.

First, oxygen must be made more readily available and accessible at health care facilities providing care to adults, with emphasis being placed on public and lower-tier health centers. Stakeholder engagement is key to this process and needs assessments should be done to ensure facilities and communities are involved in decision-making. This will require a concerted effort by national, regional and local governments, ministries of health, policy experts, health care workers, and health facility leadership to identify the appropriate oxygen delivery system for each setting and to ensure adequate resources are available for the maintenance of these systems.

Our analysis shows that concentrators are more cost-effective than oxygen cylinders in areas where there is reliable access to power. Innovation around lowering the cost of devices and providing reservoirs during outages may be helpful, and there will need to be a significant focus on building infrastructure around reliable power. Projects in resource-limited settings outside of SSA may be helpful in this regard. For example, studies in Papua New Guinea describe the process and effectiveness of an oxygen program which included provision of pulse oximeters, training of staff and installation of oxygen concentrators, as well as the design and feasibility of a solar-powered oxygen system [48,49]. Similarly, trials focused on improving access to oxygen among pediatric populations in SSA, especially those exploring solar-power as an energy source, may provide the data and basic infrastructure needed to improve access for adults [50-52]. Innovations such as these are critical in facilitating oxygen availability in lower-tier health centers.

Second, health care workers need appropriate equipment, education, training, and feedback in order to use oxygen appropriately and effectively. Identifying patients who need oxygen therapy is limited by a lack of pulse oximeters and knowledge, and appropriately initiating, titrating, and discontinuing oxygen therapy is limited by a lack of knowledge and training among health care workers at all levels. Pulse oximeters should be in place at every health care facility. They are an easy-to-use and relatively affordable method of identifying patients at greatest risk of mortality. Oxygen tubing and patient delivery devices such as nasal prongs or face masks must be readily available, and a steady supply chain must be maintained. Education and training of health care workers around oxygen saturation, hypoxemia, and effective oxygen dosing can ensure that resources are being used efficiently [44].

A challenge in administering oxygen not yet addressed in this review is the oxygen saturation threshold below which oxygen should be provided in various settings and among various populations. Most included studies used an oxygen saturation below 90% as their threshold. This is reasonable for most patients with acute hypoxemia and is the threshold recommended in a WHO manual on oxygen therapy for children [53]. However, for patients with chronic hypoxemia from primary lung disease or for populations living at higher altitude where the partial pressure of oxygen is lower, a lower threshold may be more appropriate. These populations often have physiologic compensations allowing them to tolerate lower oxygen levels; providing excess oxygen may in fact cause harm. Therefore, education and training has to be contextualized for each setting and practitioners need to be capable of individualizing therapy for each patient.

Third, there were no studies assessing the prevalence of hypoxemia among outpatients and the availability of long-term oxygen therapy for home use. If we extrapolate from the severe limitations in hospital settings, we can infer this infrastructure is severely limited. In 2017, over 544 million people had a chronic respiratory disease, representing an increase of nearly 40% since 1990 [54]. Prevalence in SSA is likely underestimated due to a lack of diagnostic capabilities, but is expected to grow as life-expectancy increases in many countries [55]. Any efforts to reduce disability or mortality from chronic respiratory disease that do not include building infrastructure for long-term oxygen therapy will fall short given what is known about its mortality and quality of life benefits [3,4].

Finally, while this review was conceptualized and undertaken prior to the COVID-19 pandemic, it is impossible to ignore the disparities in resource allocation that the pandemic has underscored. Articles in the popular media have highlighted the enormous need for oxygen in countries across the world, many in SSA, as well as the incredible barriers in accessing it [56,57]. This enhanced awareness has facilitated increased work and innovation in this area [58]. As donations of oxygen concentrators pour in from international organizations and aid agencies, it will be important to ensure that aid extends past simple provision of these systems to maintenance, training, and local capacity building.

The major strength of our scoping review is its comprehensive scope and wide inclusion of studies addressing oxygen delivery systems in various ways. We aimed to evaluate the depth and breadth of knowledge and research on oxygen delivery systems in SSA and were able to summarize the evidence in this field. Limitations of our study include that this is not a systematic review, and therefore we cannot aim to assess the quality of articles or make definitive inferences; similarly, we cannot aim to assess the risk of bias given the descriptive nature of our objectives and the types of studies presented which were mainly surveys and observational data; by limiting our review to full-text articles, we may have missed relevant data available in abstracts; and included studies encompassed only 22 of 46 SSA countries so may not be representative of the general population or region.

In conclusion, our findings highlight the substantial need for further research and building capacity for oxygen therapy for adults across SSA and signals a call to action. We provide multiple potential action items for health care workers, researchers, policy makers, and organizations to consider as we move towards improving the care of and outcomes among patients with respiratory diseases.

## References

[1] Societies FoIR. The Global Impact of Respiratory Disease – Second Edition. Sheffield, European Respiratory Society; 2017.

[2] Heffner JE The Story of Oxygen. Respir Care. 2013;58:18-31. 10.4187/respcare.01831 23271817

[3] Nocturnal Oxygen Therapy Trial Group Continuous or nocturnal oxygen therapy in hypoxemic chronic obstructive lung disease: a clinical trial. Ann Intern Med. 1980;93:391-8. 10.7326/0003-4819-93-3-391 6776858

[4] Report of the Medical Research Council Working Party Long term domiciliary oxygen therapy in chronic hypoxic cor pulmonale complicating chronic bronchitis and emphysema. Lancet. 1981;1:681-6. 6110912

[5] World Health Organization. WHO Model List of Essential Medicines. 2017. Available: https://apps.who.int/iris/bitstream/handle/10665/273826/EML-20-eng.pdf?ua=1. Accessed: 28 October 2019.

[6] World Health Organization. WHO Model List of Essential Medicines. 2002. Available: https://apps.who.int/iris/bitstream/handle/10665/67335/a76618.pdf?sequence=1. Accessed: 28 October 2019.

[7] Technical specifications for oxygen concentrators. Geneva: World Health Organization; 2015.

[8] World Health Organization. Oxygen therapy for children. 2016. Available: https://apps.who.int/iris/bitstream/handle/10665/204584/9789241549554_eng.pdf?sequence=1. Accessed: 28 October 2019.

[9] Arksey H O’Malley L Scoping studies: towards a methodological framework. Int J Soc Res Methodol. 2005;8:19-32. 10.1080/1364557032000119616

[10] Levac D Colquhoun H O'Brien KKJI Scoping studies: advancing the methodology. 2010;5:69. 10.1186/1748-5908-5-69 PMC2954944 20854677

[11] Cochrane Effective Practice and Organisation of Care. Data collection form. EPOC Resources for review authors. 2017. Available: epoc.cochrane.org/epoc-resources-review-authors. Accessed: 20 April 2019.

[12] Adipa FE Aziato L Zakariah AN Qualitative exploration of nurses’ perspectives on clinical oxygen administration in Ghana. Int J Afr Nurs Sci. 2015;2:42-6. 10.1016/j.ijans.2015.03.002

[13] Albutt K Punchak M Kayima P Namanya DB Anderson GA Shrime MG Access to Safe, Timely, and Affordable Surgical Care in Uganda: A Stratified Randomized Evaluation of Nationwide Public Sector Surgical Capacity and Core Surgical Indicators. World J Surg. 2018;42:2303-13. 10.1007/s00268-018-4485-1 29368021

[14] Albutt K Drevin G Yorlets RR Svensson E Namanya DB Shrime MG ‘We are all serving the same Ugandans’: A nationwide mixed-methods evaluation of private sector surgical capacity in Uganda. PLoS One. 2019;14:e0224215. 10.1371/journal.pone.0224215 31648234 PMC6812829

[15] Belle J Cohen H Shindo N Lim M Velazquez-Berumen A Ndihokubwayo JB Influenza preparedness in low-resource settings: a look at oxygen delivery in 12 African countries. J Infect Dev Ctries. 2010;4:419-24. 10.3855/jidc.859 20818088

[16] Bradley BD Chow S Nyassi E Cheng YL Peel D Howie SRC A retrospective analysis of oxygen concentrator maintenance needs and costs in a low-resource setting: experience from The Gambia. Health Technol (Berl). 2015;4:319-28. 10.1007/s12553-015-0094-2

[17] Bradley BD Light JD Ebonyi AO N’Jai PC Ideh RC Ebruke BE Implementation and 8-year follow-up of an uninterrupted oxygen supply system in a hospital in The Gambia. Int J Tuberc Lung Dis. 2016;20:1130-4. 10.5588/ijtld.15.0889 27393551 PMC4937752

[18] Burssa D Teshome A Iverson K Ahearn O Ashengo T Barash D Safe Surgery for All: Early Lessons from Implementing a National Government-Driven Surgical Plan in Ethiopia. World J Surg. 2017;41:3038-45. 10.1007/s00268-017-4271-5 29030677

[19] Calderon R Morgan MC Kuiper M Nambuya H Wangwe N Somoskovi A Assessment of a storage system to deliver uninterrupted therapeutic oxygen during power outages in resource-limited settings. PLoS One. 2019;14:e0211027. 10.1371/journal.pone.0211027 30726247 PMC6364892

[20] Desalu OO Onyedum CC Iseh KR Salawu FK Salami AK Asthma in Nigeria: are the facilities and resources available to support internationally endorsed standards of care? Health Policy. 2011;99:250-4. 10.1016/j.healthpol.2010.10.006 21056506

[21] Evans HT Mahmood N Fullerton DG Rylance J Gonani A Gordon SB Oxygen saturations of medical inpatients in a Malawian hospital: cross-sectional study of oxygen supply and demand. Pneumonia. 2016;1:3-6. Nathan Qld. 10.15172/pneu.2012.1/208 31463177 PMC6707393

[22] Hill SE Njie O Sanneh M Jallow M Peel D Njie M Oxygen for treatment of severe pneumonia in The Gambia, West Africa: a situational analysis. Int J Tuberc Lung Dis. 2009;13:587-93. 19383191

[23] Howie SR Hill SE Peel D Sanneh M Njie M Hill PC Beyond good intentions: lessons on equipment donation from an African hospital. Bull World Health Organ. 2008;86:52-6. 10.2471/BLT.07.042994 18235890 PMC2647344

[24] Howie SR Hill S Ebonyi A Krishnan G Njie O Sanneh M Meeting oxygen needs in Africa: an options analysis from the Gambia. Bull World Health Organ. 2009;87:763-71. 10.2471/BLT.08.058370 19876543 PMC2755310

[25] Kouo-Ngamby M Dissak-Delon FN Feldhaus I Juillard C Stevens KA Ekeke-Monono M A cross-sectional survey of emergency and essential surgical care capacity among hospitals with high trauma burden in a Central African country. BMC Health Serv Res. 2015;15:478. 10.1186/s12913-015-1147-y 26496762 PMC4619297

[26] Kushner AL Cherian MN Noel L Spiegel DA Groth S Etienne C Addressing the Millennium Development Goals from a surgical perspective: essential surgery and anesthesia in 8 low- and middle-income countries. Arch Surg. 2010;145:154-9. 10.1001/archsurg.2009.263 20157083

[27] LeBrun DG Chackungal S Chao TE Knowlton LM Linden AF Notrica MR Prioritizing essential surgery and safe anesthesia for the Post-2015 Development Agenda: Operative capacities of 78 district hospitals in 7 low- and middle-income countries. Surgery. 2014;155:365-73. 10.1016/j.surg.2013.10.008 24439745

[28] Nyarko KM Ameme DK Ocansey D Commeh E Markwei MT Ohene SA Capacity assessment of selected health care facilities for the pilot implementation of Package for Essential Non-communicable Diseases (PEN) intervention in Ghana. Pan Afr Med J. 2016;25:16. 10.11604/pamj.supp.2016.25.1.6252 28149441 PMC5257011

[29] Ologunde R Vogel JP Cherian MN Sbaiti M Merialdi M Yeats J Assessment of cesarean delivery availability in 26 low- and middle-income countries: a cross-sectional study. Am J Obstet Gynecol. 2014;211:504.e1-.e12. 10.1016/j.ajog.2014.05.022 24844851

[30] Otiangala D Agai NO Olayo B Adudans S Ng CH Calderon R Oxygen insecurity and mortality in resource-constrained healthcare facilities in rural Kenya. Pediatr Pulmonol. 2020;55:1043-9. 10.1002/ppul.24679 32040889

[31] Opio MO Nansubuga G Kellett J In-hospital mortality of acutely ill medical patients admitted to a resource poor hospital in sub-Saharan Africa and to a Canadian regional hospital compared using the abbreviated VitalPAC Early Warning Score. Eur J Intern Med. 2014;25:142-6. 10.1016/j.ejim.2013.09.013 24140259

[32] Ouedraogo NS Schimanski C Energy poverty in healthcare facilities: a “silent barrier” to improved healthcare in sub-Saharan Africa. J Public Health Policy. 2018;39:358-71. 10.1057/s41271-018-0136-x 29950575

[33] Penoyar T Cohen H Kibatala P Magoda A Saguti G Noel L Emergency and surgery services of primary hospitals in the United Republic of Tanzania. BMJ Open. 2012;2:e000369. 10.1136/bmjopen-2011-000369 22307096 PMC3274714

[34] Rassool RP Sobott BA Peake DJ Mutetire BS Moschovis PP Black JF A Low-Pressure Oxygen Storage System for Oxygen Supply in Low-Resource Settings. Respir Care. 2017;62:1582-7. 10.4187/respcare.05532 28951467 PMC6373847

[35] Rudd KE Tutaryebwa LK West TE Presentation, management, and outcomes of sepsis in adults and children admitted to a rural Ugandan hospital: A prospective observational cohort study. PLoS One. 2017;12:e0171422. 10.1371/journal.pone.0171422 28199348 PMC5310912

[36] Aston SJ Ho A Jary H Huwa J Mitchell T Ibitoye S Etiology and Risk Factors for Mortality in an Adult Community-acquired Pneumonia Cohort in Malawi. Am J Respir Crit Care Med. 2019;200:359-69. 10.1164/rccm.201807-1333OC 30625278 PMC6680311

[37] Dickson SJ Clay KA Adam M Ardley C Bailey MS Burns DS Enhanced case management can be delivered for patients with EVD in Africa: Experience from a UK military Ebola treatment centre in Sierra Leone. J Infect. 2018;76:383-92. 10.1016/j.jinf.2017.12.006 29248587 PMC5903873

[38] Foran M Ahn R Novik J Tyer-Viola L Chilufya K Katamba K Prevalence of undiagnosed hypoxemia in adults and children in an under-resourced district hospital in Zambia. Int J Emerg Med. 2010;3:351-6. 10.1007/s12245-010-0241-5 21373304 PMC3047821

[39] Hesse IF Knowledge of asthma and its management in newly qualified doctors in Accra, Ghana. Respir Med. 1995;89:35-9. 10.1016/0954-6111(95)90068-3 7708978

[40] Kabeza AB Twagirumugabe T Walker I Shigemasa I Incidence and risk factors of undetected postoperative hypoxaemia at a Teaching Hospital in AfricaRwanda: The usefulness of Portable oximeter. Br J Anaesth. 2012;108:ii314.

[41] Mwita CC Muthoka J Maina S Mulingwa P Gwer S Early management of traumatic brain injury in a Tertiary hospital in Central Kenya: A clinical audit. J Neurosci Rural Pract. 2016;7:97-101. 10.4103/0976-3147.165390 26933354 PMC4750351

[42] Sani MU Cotter G Davison BA Mayosi BM Damasceno A Edwards C Symptoms and Signs of Heart Failure at Admission and Discharge and Outcomes in the Sub-Saharan Acute Heart Failure (THESUS-HF) Registry. J Card Fail. 2017;23:739-42. 10.1016/j.cardfail.2016.09.016 27664511

[43] Scott JW Nyinawankusi JD Enumah S Maine R Uwitonze E Hu Y Improving prehospital trauma care in Rwanda through continuous quality improvement: an interrupted time series analysis. Injury. 2017;48:1376-81. 10.1016/j.injury.2017.03.050 28420542

[44] Sutherland T Moriau V Niyonzima JM Mueller A Kabeja L Twagirumugabe T The “Just Right” Amount of Oxygen. Improving Oxygen Use in a Rwandan Emergency Department. Ann Am Thorac Soc. 2019;16:1138-42. 10.1513/AnnalsATS.201811-763QI 31145642

[45] Toure NO Diao M Kane A Diop IB Sarr M Ba SA Chronic cor pulmonale: a study of 34 cases in the Dakar University Hospital Center Cardiology Department. Dakar Méd. 2000;45:108-12. 15779162

[46] Worodria W Chang E Andama A Sanyu I Byanyima P Musisi E Predictors of Mortality Among Hospitalized Patients With Lower Respiratory Tract Infections in a High HIV Burden Setting. J Acquir Immune Defic Syndr. 2018;79:624-30. 10.1097/QAI.0000000000001855 30222660 PMC6231969

[47] Salami TA Exacerbators and treatment outcomes of hospitalised patients with chronic obstructive pulmonary disease (COPD) in a suburban centre in Nigeria. Am J Respir Crit Care Med. 2017;195:A1700.

[48] Matai S Peel D Wandi F Jonathan M Subhi R Duke TJA Implementing an oxygen programme in hospitals in Papua New Guinea. Ann Trop Paediatr. 2008;28:71-8. 10.1179/146532808X270716 18318953

[49] Duke T Hwaihwanje I Kaupa M Karubi J Panauwe D Sa’avu M Solar powered oxygen systems in remote health centers in Papua New Guinea: a large scale implementation effectiveness trial. J Global Health. 2017;7:010411. 10.7189/jogh.07.010411 PMC5441450 28567280

[50] Conradi N Mian Q Namasopo S Conroy AL Hermann LL Olaro C Solar-powered oxygen delivery for the treatment of children with hypoxemia: protocol for a cluster-randomized stepped-wedge controlled trial in Uganda. Trials. 2019;20:679. 10.1186/s13063-019-3752-2 31805985 PMC6896330

[51] Hawkes MT Conroy AL Namasopo S Bhargava R Kain KC Mian Q Solar-Powered Oxygen Delivery in Low-Resource Settings: A Randomized Clinical Noninferiority Trial. JAMA Pediatr. 2018;172:694-6. 10.1001/jamapediatrics.2018.0228 29800014 PMC6137508

[52] Turnbull H Conroy A Opoka RO Namasopo S Kain KC Hawkes M Solar-powered oxygen delivery: proof of concept. Int J Tuberc Lung Dis. 2016;20:696-703. 10.5588/ijtld.15.0796 27084827

[53] World Health Organization. Oxygen therapy for children: a manual for health workers. 2016.

[54] GBD 2017 Disease and Injury Incidence and Prevalence Collaborators Global, regional, and national incidence, prevalence, and years lived with disability for 354 diseases and injuries for 195 countries and territories, 1990–2017: a systematic analysis for the Global Burden of Disease Study 2017. Lancet. 2018;392:1789-858. 10.1016/S0140-6736(18)32279-7 30496104 PMC6227754

[55] Labaki WW Han MK Chronic respiratory diseases: a global view. Lancet Respir Med. 2020;8:531-3. 10.1016/S2213-2600(20)30157-0 32526184 PMC8034823

[56] McNeil DGJ. A Simple Way to Save Lives as Covid-19 Hits Poorer Nations. The New York Times. 2020 2020/09/30/T12:44:43-04:00;Sect. Health.

[57] Davies MMS, Onwuzoo A. Lack of oxygen leaves patients in Africa gasping for air. The Bureau of Investigative Journalism. Available: https://www.thebureauinvestigates.com/stories/2020-08-09/lack-of-oxygen-leaves-covid-19-patients-in-africa-gasping-for-air. Accessed: 9 August 2020.

[58] Canada G. Innovators mobilize to help developing countries combat Covid-19. 2020. Available: https://www.grandchallenges.ca/2020/innovators-mobilize-to-help-developing-countries-combat-covid-19/. Accessed: 15 February 2020.

